# Grazing Prevalence and Associations with Eating and General Psychopathology, Body Mass Index, and Quality of Life in a Middle-Income Country

**DOI:** 10.3390/nu15030557

**Published:** 2023-01-20

**Authors:** Dean Spirou, Andreea I. Heriseanu, Rosely Sichieri, Phillipa Hay, Carlos E. Moraes, Jose C. Appolinario

**Affiliations:** 1 School of Medicine, Western Sydney University, Sydney, NSW 2214, Australia; 2 Blacktown Metabolic and Weight Loss Program, Department of Endocrinology and Diabetes, Blacktown Hospital, Sydney, NSW 2148, Australia; 3 eCentreClinic, School of Psychological Sciences, Macquarie University, Macquarie Park, NSW 2109, Australia; 4 Social Medicine Institute, State University of Rio de Janeiro, Rio de Janeiro 22290-140, Brazil; 5 Translational Health Research Institute, Western Sydney University, Sydney, NSW 2560, Australia; 6 Mental Health Services, SWSLHD, Campbelltown, NSW 2560, Australia; 7 Obesity and Eating Disorders Group, Institute of Psychiatry, Federal University of Rio de Janeiro, Rio de Janeiro 22290-140, Brazil

**Keywords:** grazing, eating disorders, body mass index, depression, anxiety, quality of life, middle-income country, epidemiology, public health, eating behaviours

## Abstract

Research from high-income countries has shown that grazing is a common but problematic eating pattern, particularly when associated with a sense of loss of control. However, it is unclear whether these patterns hold globally. Thus, the goal of this study was to extend previous research by examining the prevalence and clinical correlates of compulsive grazing (CG) and non-compulsive grazing (NCG) in a middle-income country. Participants (*N* = 2297) comprised adult residents from Rio de Janeiro, Brazil. Recruitment of this population-based household survey occurred from September 2019 to February 2020. The short inventory of grazing was used to operationalise grazing subtypes. Chi-square analyses, logistic regression, and univariate tests were conducted using the complex samples procedure. The point prevalence of regular CG was 10.2% (*n* = 239) and was consistent with high-income countries, while NCG was 29.8% (*n* = 679) and was less frequent than reported in high-income countries. Additionally, similar to high-income countries, CG was associated with a higher body mass index and higher odds of eating disorders, eating disorder symptomatology, depression, anxiety, and a lower physical and mental health-related quality of life, than no grazing and NCG. Overall, this study demonstrated that grazing patterns in high-income countries extend to middle-income countries.

## 1. Introduction

Eating disorders are a prevalent global health concern [1]. Eating disorders contribute to increased mortality, disability, physical health problems, impaired psychosocial functioning, and a reduced quality of life [2,3]. Recent literature has highlighted the importance of examining the full spectrum of eating patterns (e.g., grazing), even those not formally recognised as an eating disorder in the Diagnostic and Statistical Manual of Mental Disorders (DSM-5) [2] and the International Statistical Classification of Diseases and Related Health Problems (ICD-11) [4].

Grazing is an eating pattern that has received recent attention in the clinical literature [5]. Grazing is defined as the unplanned and repetitive consumption of small amounts of food, not related to hunger sensations [6]. Two subtypes of grazing have been suggested: a non-compulsive and compulsive subtype, although other dimensional classification schemes have also been proposed [6]. Non-compulsive grazing (NCG) refers to eating in a distracted manner across an extended period, while compulsive grazing (CG) is characterised by a sense of loss of control and an inability to resist eating [6].

Previous research has demonstrated that grazing is common in people with obesity and eating disorders as well as non-clinical populations. Heriseanu et al. [5] found that the mean pooled prevalence of grazing was 33.2% in treatment-seeking patients with obesity and 23.3% in community participants with a high body mass index (BMI ≥ 30). In addition, they found that 67.8% of individuals with binge-eating disorder (BED) and 58.3% of individuals with bulimia nervosa (BN) reported grazing [5]. In university and community samples, grazing, and grazing-like eating patterns (e.g., picking, nibbling) have been found to be very common [7,8], though the precise prevalence is uncertain due to varying definitions, which encompass various types of eating behaviours. Furthermore, in an epidemiological study of grazing in the Australian population, 38.0% of participants reported regular NCG and 10.2% reported regular CG [9].

Critically, the CG subtype has been associated with adverse clinical features. In non-clinical samples, individuals with CG demonstrated significantly higher eating disorder psychopathology, higher psychological and grazing-related distress, and a lower mental health-related quality of life (MHRQoL) than those with NCG [7,10,11]. In clinical samples, bariatric surgery candidates with CG demonstrated an elevated psychopathology (e.g., depression, anxiety) and more disordered eating behaviours than individuals with NCG [12]. Similarly, in pre-bariatric surgery patients, grazing was significantly correlated with eating disorder psychopathology, disordered eating, and psychological distress, as well as reduced weight loss and increased weight regain post-surgery [10]. Further, in an epidemiological study of grazing, individuals with CG exhibited significantly higher odds of eating disorder psychopathology, higher odds of belonging to an eating disorder diagnostic group, a lower MHRQoL, and higher BMI than those with NCG or no grazing (NG) [9]. 

Despite the growing global prevalence of eating disorders and obesity, and the increasing attention on emerging disordered eating behaviours, such as grazing [5,13], the majority of literature on grazing is derived from high-income countries. Consequently, the only epidemiological study on the prevalence and correlates of grazing was from Australia, a high-income country [9]. Although a recent study [14] examined the prevalence and correlates of several DSM-5 eating disorders (e.g., BED, BN) in a representative sample from Brazil, a middle-income country, there have been no population-based studies on grazing in a middle-income country. As a result, it is unclear whether grazing patterns from high-income countries also hold true in lower-income countries. This is an important clinical question because grazing patterns may vary across socioeconomically diverse populations. In addition, we cannot draw conclusions about global health epidemiology and develop health policies based exclusively on high-income countries. Therefore, the goal of the current study was to elaborate on previous research by examining the prevalence of grazing in a representative sample of residents from Rio de Janeiro, Brazil. Additionally, this study aimed to examine the relationship between grazing subtypes and eating disorders, eating disorder symptomatology, BMI, psychological difficulties, and health-related quality of life (HRQoL) in this middle-income population. This study extends previous research on the clinical correlates of grazing by controlling for potentially confounding sociodemographic variables and employing comprehensive and multi-method assessment procedures to evaluate eating disorder diagnoses.

## 2. Method

### 2.1. Participants and Design

The current study was part of the broader Binge-Eating in Rio survey developed for the general population of Rio de Janeiro, Brazil. This study was designed as an in-person, population-based, household survey. The survey was developed according to a clustered and stratified probability sample, with selection occurring in a 3-stage process: census enumeration areas, household selection, and participant selection (see [14] for more information). Eligible participants comprised adults aged 18 to 60 years residing in Rio de Janeiro, Brazil. Breastfeeding and pregnant women were excluded. All participants provided written, informed consent. This study was approved by the Federal University of Rio de Janeiro, Institute of Psychiatry (CAAE 03814818.7.0000.5263).

### 2.2. Procedure

Data collection occurred in two phases from September 2019 to February 2020. During phase 1, interviewers invited each selected household to participate in a survey on their mental health and eating behaviours. Of the 2985 eligible households, 688 declined to participate, resulting in 2297 households enrolling (77% participation rate). Subsequently, an eligible adult from each enrolled household was selected to participate. Trained interviewers measured the selected participants’ height and weight and invited them to complete a series of research questionnaires on a tablet. In phase 2, a randomly selected subgroup of participants that screened negative for BED and BN, and all participants that screened positive, were invited to complete an interview via telephone to confirm diagnoses (see [14] for more information).

## 3. Measures 

### 3.1. Sociodemographics

Sociodemographic characteristics including age, gender, marital status, race/ethnicity, employment status, education, and income were self-reported by participants.

### 3.2. Anthropometrics

Participant height was measured on a portable stadiometer (model 206; Seca^®^, Hamburg, Germany), while weight was measured on a digital scale (Plenna^®^, São Paulo, Brazil). BMI was calculated according to weight and height measurements (kg/m^2^).

### 3.3. Short Inventory of Grazing (SIG)

The SIG is a 2-item self-report measure of grazing frequency over the last three months [7]. Item 1 assesses grazing in general (i.e., NCG), while item 2 assesses grazing characterised by a sense of loss of control (i.e., CG). Items are rated on a 7-point Likert scale from 0 (none at all) to 6 (eight or more times per week). Higher scores indicate a higher grazing frequency. In this study, three grazing subtypes were established: regular CG (grazing at least once per week with a loss of control), regular NCG (grazing at least once per week without a loss of control), and NG (none or less than weekly grazing). Participants that reported both NCG and CG were classified into the CG group to maintain independence between the categories. The SIG has demonstrated acceptable psychometric properties in an Australian normal weight sample [7] and has been cross-culturally adapted and validated in a Brazilian sample [15]. 

### 3.4. Questionnaire on Eating and Weight Patterns-5 (QEWP-5)

The QEWP-5 is a 26-item self-report screening measure of BED and BN [16]. A Brazilian-Portuguese version of the QEWP-5 was utilised in this study [17]. The QEWP-5 assesses participant demographics (e.g., age, gender), anthropometrics (i.e., weight and height), objective binge-eating (OBE) and subjective binge-eating (SBE) including frequency, duration, and the associated distress and compensatory behaviours (e.g., laxative/diuretic misuse), body weight and shape evaluation, and parents’ silhouettes. The QEWP-5 also provides a possible diagnosis of BED and BN according to the DSM-5 criteria. In this study, a dichotomous approach was used to identify the presence or absence of BED and BN. The Brazilian-Portuguese version of QEWP-5 has demonstrated reliability and validity in the assessment of disordered eating behaviours in the general population of Brazil [18,19].

### 3.5. Patient Health Questionnaire-9 (PHQ-9)

The PHQ-9 is a 9-item self-report measure of depressive symptoms in the last two weeks [20]. A Brazilian-validated version of the PHQ-9 was utilised in this study [21]. Items are rated on a 4-point Likert scale from 0 (not at all) to 3 (nearly every day). Each item corresponds with a symptom of major depression in the DSM-IV. The total score ranges from 0 to 27, with higher scores indicating more severe depression. Depression severity is indicated using cut-off scores of 5 (mild), 10 (moderate), 15 (moderately severe), and 20 (severe). In this study, depression was identified using a cut-off score ≥10, which has shown high specificity and sensitivity for detecting major depression [22]. The PHQ-9 has demonstrated acceptable psychometric properties and is a valid and reliable measure of depression severity [22].

### 3.6. Generalised Anxiety Disorder-7 (GAD-7)

The GAD-7 is a 7-item self-report measure of generalised anxiety disorder (GAD) symptoms in the last two weeks [23]. A Brazilian-Portuguese version of the GAD-7 was utilised in this study [24]. Items are rated on a 4-point Likert scale from 0 (not at all) to 3 (nearly every day). Items primarily correspond with DSM-IV symptoms of GAD. The total score ranges from 0 to 21, with higher scores indicating more severe anxiety. Anxiety severity is indicated using cut-off scores of 5 (mild), 10 (moderate), and 15 (severe). In this study, anxiety was identified using a cut-off score ≥10, which has been shown as a reasonable cut-off for detecting GAD [23]. The GAD-7 has demonstrated good validity and reliability, and is a valid instrument for identifying GAD severity [23].

### 3.7. 12-Item Short-Form Health Survey (SF-12)

The SF-12 is a 12-item self-report measure of the HRQoL [25]. The SF-12 comprises two scales: physical component summary (PCS) and mental component summary (MCS). The PCS provides an indication of the physical HRQoL (PHRQoL), while the MCS reflects the MHRQoL. Higher scores indicate better physical and mental functioning. The SF-12 has demonstrated satisfactory validity and reliability in a Brazilian sample [26].

## 4. Statistical Analyses

All statistical analyses were conducted using the complex samples procedure in IBM, New York, NY, USA, SPSS Version 29.0. Prior to analyses, data were weighted to account for variations in the probability of selection at different stages of the recruitment process and non-responses. The complex samples procedure performs statistical analyses with consideration of these weights and the complex design of the survey. The point prevalence (%) of each grazing subtype was calculated using the weighted frequencies. The number of participants in each group (*n*) were calculated using the unweighted count. Chi-square (χ^2^) analyses were conducted to examine the frequencies (*n*, %) and differences in sociodemographic characteristics, eating disorders, eating disorder symptomatology, and psychological difficulties across grazing subtypes. Significance, odds ratio (OR), and 95% confidence intervals (CIs) were computed for chi-square analyses according to the weighted values. OR was only calculated for outcome variables in 2-by-2 tables (i.e., eating disorders, eating disorder symptomatology, and psychological difficulties). OR is the most used index of effect size in epidemiological research [27]. Logistic regression was used to follow-up significant chi-square analyses and to control for sociodemographic differences across grazing subtypes. T-tests were conducted using the general linear model complex samples procedure to examine differences in the HRQoL, age, and BMI across grazing subtypes. The alpha level was set to 0.05 across all analyses, with the Bonferroni correction applied to analyses of outcome variables.

## 5. Results 

### 5.1. Sociodemographic Characteristics

In the final sample (*N* = 2297), approximately 52% of the participants identified as women and 48% as men. The mean participant age was 38.18 years (*SE* = 0.44), and the mean BMI was 27.61 kg/m^2^ (*SE* = 0.19). Most participants were from mixed race/ethnicity (43.2%). The median education level was 11–14 years (46.3%; equivalent to high school). The median monthly income was 1001–3000 BRL (50.3%), reflecting the current median income distribution in the general population of Brazil (equivalent to 190–568 USD). Most participants were in paid employment (63.5%), and most were married (54.2%).

### 5.2. Grazing and Sociodemographic Characteristics

According to the SIG, the point prevalence of regular CG was 10.2% (95% CI [8.5, 12.3]; *n* = 239), while the point prevalence of regular NCG was 29.8% (95% CI [26.1, 33.8]; *n* = 679). The remaining 1371 (60.0%) participants reported none or less than weekly grazing (i.e., NG). There were significant differences in terms of gender distribution, age, and occupational status, such that more women than men engaged in CG, those engaging in any type of grazing were younger, and those in paid employment engaged in both types of grazing more than those in other occupational groups. Table 1 presents the sociodemographic and weight characteristics of the final sample according to grazing subtype (see Supplementary Table S1 for additional test statistics).

### 5.3. Grazing and BMI

There were significant differences between the CG and NG group on BMI class and obesity class, and the CG and NCG group on BMI class. Specifically, individuals with CG had significantly higher BMI than those with NG and those with NCG. However, there was no significant difference in BMI between the NCG and NG group. These results remained significant after controlling for sociodemographic differences (see Table 1).

### 5.4. Grazing and Eating Disorders

Individuals with CG had significantly higher odds of having a BED or BN diagnosis than those with NG and those with NCG. However, those with NCG did not significantly differ from those with NG on BED and BN. These results remained significant after controlling for sociodemographic differences. Table 2 presents the prevalence and OR of eating disorders and eating disorder symptomatology according to grazing subtype.

### 5.5. Grazing and Eating Disorder Symptomatology

Individuals with CG demonstrated significantly higher odds of engaging in OBE and SBE than those with NG and those with NCG. Individuals with CG also had significantly higher odds of compensatory behaviours following SBE than those with NG and those with NCG. However, there was no significant difference in compensatory behaviours following OBE among those with CG and NG, and those with CG and NCG after controlling for sociodemographic differences. Further, those with CG had significantly higher odds of body dissatisfaction and weight/shape overvaluation than those with NG, while those with CG had significantly higher odds of body dissatisfaction but not weight/shape overvaluation compared to those with NCG (see Table 2).

Individuals with NCG had significantly higher odds of SBE, weight/shape overvaluation, and body dissatisfaction than those with NG. However, there was no significant difference between those with NCG and NG on OBE and compensatory behaviours following both OBE and SBE. These results remained significant after controlling for sociodemographic differences (see Table 2).

### 5.6. Grazing and Psychological Difficulties

Individuals with CG had significantly higher odds of a clinical level of depression and anxiety than those with NG and those with NCG using the dichotomous cut-off scores on the PHQ-9 and GAD-7, respectively. In addition, those with NCG had significantly higher odds of anxiety than those with NG, while there was no significant difference between the NCG and NG groups in depression after controlling for sociodemographic differences. Table 3 presents the prevalence and OR of the psychological difficulties according to grazing subtype.

### 5.7. Grazing and HRQoL

Individuals with CG had significantly lower scores on the SF-12-PCS, SF-12-MCS, and SF-12 total scores compared to those with NG and NCG. Further, individuals with NCG had significantly lower scores on the MCS and total score than those with NG. However, there was no significant difference between the NCG and NG groups on the PCS. These results remained significant after controlling for sociodemographic differences. Table 4 presents the means, standard error, and 95% CIs for the HRQoL according to grazing subtype (see Supplementary Table S1 for additional test statistics).

## 6. Discussion

This study was the first to examine the epidemiology of grazing in a representative sample from a middle-income country. First, our results revealed that 10.2% of the sample engaged in regular CG. This is consistent with the findings of Heriseanu et al. [9], who also reported that 10.2% of their high-income sample engaged in CG. In addition, we found that 29.8% of the sample engaged in regular NCG. This frequency is relatively lower than the findings of Heriseanu et al. [9], who reported that 38.0% of their high-income sample engaged in NCG. One explanation for the discrepancy in NCG frequency may be related to socioeconomic factors. For instance, Heriseanu et al. [9] reported that grazing was associated with residing in a metropolitan area and higher-income households, which are indices of higher socioeconomic status. In contrast, the current study was conducted in a middle-income region with high levels of socioeconomic disadvantage [14]. Previous research has found that socioeconomic factors, including the affordability, accessibility, and convenience of food, may influence food selection in middle-income countries, such as Brazil [28,29]. Indeed, health perception (e.g., perceived health benefits) and sensory appeal (e.g., taste) as well as sociodemographic (e.g., age, gender), psychological (e.g., mood), and sociocultural factors (e.g., culture, religion, traditions, food beliefs) may also impact food selection in middle-income populations [28,29]. Thus, there are several factors that drive food choices in residents from middle-income regions, some of which may limit their ability to engage in generic grazing, relative to those from high-income areas. 

Conversely, the finding that CG was uniform across middle- and high-income populations is consistent with previous population-based research in Australia, which found that eating disorder symptoms are dispersed equally across socioeconomic levels [30]. This may be because disordered eating patterns, such as CG, supersede socioeconomic and sociocultural barriers due to the clinical severity of this behaviour. On the other hand, NCG does not represent a disordered level of grazing and is therefore likely to be impacted by socioeconomic factors, such as income. Taken together, these results indicate that the prevalence of CG is consistent across middle- and high-income populations, while NCG appears to be less frequent in middle-income regions.

Second, consistent with previous research [7,9], individuals with CG had a significantly higher BMI than those with NG or NCG. Additionally, our results indicated that almost half of the participants in the CG group had a BMI over 30 kg/m^2^. This frequency is similar to previous studies in the literature, with a prevalence ranging between 23.3% and 55.7% [5]. While most research has been conducted in treatment-seeking patients with a high BMI (e.g., bariatric surgery clinics) and in high-income countries, the current findings suggest that CG may contribute to weight gain or weight management problems in the general population for both middle- and high-income samples, highlighting the importance of targeted clinical attention for this eating behaviour.

Third, after controlling for sociodemographic differences, our results indicated that individuals with CG demonstrated significantly higher odds of having a BED or BN diagnosis, as well as significantly higher odds of eating disorder symptomatology, than those with NG or NCG. These findings are consistent with previous epidemiological research [9] and other literature, which found significantly higher disordered eating psychopathology in clinical and non-clinical samples for those with CG relative to those with NG or NCG [7,10,11,12]. In contrast to Heriseanu et al. [9], however, we did not find increased odds of eating disorder diagnosis or symptomatology for those with NCG compared to those with NG. Overall, these findings highlight that CG is associated with increased odds of eating disorder symptomatology compared to NG or NCG in both middle- and high-income populations.

Fourth, after controlling for sociodemographic differences, our results indicated that individuals with CG demonstrated significantly higher odds of clinically significant depression and anxiety than those with NG and those with NCG. These findings are consistent with previous studies that demonstrated more severe psychological distress among those with CG relative to those with NCG [7,12]. Further, individuals with NCG had higher odds of anxiety than those with NG, a relationship not previously examined in the literature. Together, these findings suggest that CG is associated with higher odds of general psychopathology than NG or NCG in both middle- and high-income populations.

Fifth, after controlling for sociodemographic differences, individuals with CG had a significantly lower MHRQoL, PHRQoL, and overall HRQoL than those with NG or NCG. Additionally, individuals with NCG had a significantly lower MHRQoL and overall HRQoL than those with NG. These results are partially consistent with previous studies, which found a lower MHRQoL in those with CG compared to those with NG [9] or NCG [7,9]. In contrast, however, previous research found no significant difference in the MHRQoL among those with NCG or NG [9] as well as no significant difference in the PHRQoL among any grazing subtypes [5,7,9]. Individuals with CG in the current study had significantly higher BMI than those with NG or NCG. A high BMI is a risk factor for several physical health problems, including cardiovascular disease, hypertension, type 2 diabetes, musculoskeletal conditions, and some cancers [31,32]. Conde et al. [33] found that a high BMI has become a major public health concern in Brazil, and that public policies targeting obesity have been minimally effective. Unlike high-income countries, residents from middle-income countries may have more financial or geographical barriers that limit their access to resources and impact their capacity to manage the physical health problems associated with a higher BMI. Consequently, individuals from middle-income populations with CG may report a lower PHRQoL than those from high-income countries due to high BMI and socioeconomic factors, though future research is required to investigate this further.

## 7. Clinical and Public Health Implications

This study contributes to understanding the epidemiology and clinical correlates of grazing in a middle-income country. Overall, our findings suggested that the frequency of CG is consistent across middle- and high-income countries, while NCG appears to be less frequent in middle-income populations. In addition, similar to high-income populations, CG was associated with a higher BMI and higher odds of eating disorders, eating disorder symptomatology, depression, anxiety, and a lower HRQoL than NG or NCG. These findings have important clinical and public health implications.

Given that the CG frequency was consistent across middle- and high-income countries, there are significant economic and health implications for individuals in middle-income populations. For instance, those from middle-income populations will experience the same adverse clinical impacts of CG, including higher odds of mental health difficulties and a lower HRQoL, but have less access to resources and specialised services to target these concerns. This may place individuals at a heightened risk of medical and psychological complications and could result in increased economic burden and stress on public health services and local healthcare workers. To mitigate these financial and geographical barriers, it would be essential to provide more specialised public health services for eating disorders and weight management that are distributed across differing socioeconomic regions. Additionally, as proposed previously [30], to ensure equitable access to healthcare for individuals in disadvantaged areas, digital mental health treatment (i.e., telemedicine/telehealth) for eating disorder symptoms may be a viable option. Recent studies have demonstrated that telemedicine is an effective avenue for the evidence-based treatment of eating disorders in children, adolescents, and adults [34,35]. Furthermore, providing specialised and culturally nuanced training to local healthcare workers for the identification and treatment of disordered grazing patterns may circumvent economic and health consequences. 

Second, given the ubiquitous nature of grazing and the adverse clinical consequences of CG, grazing patterns should be routinely evaluated in clinical practice, especially for those presenting with an eating disorder or weight management problems. Identifying and demarcating grazing patterns may provide valuable insight into targeted clinical intervention. For example, consistent with previous research [9], we found that CG was more prevalent among individuals with a higher BMI. If, however, CG is not comprehensively assessed and treated, it may contribute to weight gain or weight management difficulties. In addition, if undetected or not directly addressed, CG may contribute to significant psychological distress (e.g., depression, anxiety) and a reduced HRQoL, even if other disordered eating behaviours are targeted. Importantly, the findings from this study support the broader clinical literature, which has suggested that it is the loss of control over grazing that primarily accounts for clinical impairment, rather than the presence of grazing itself. This reinforces the view proposed by Conceição et al. [6], that grazing presents on a continuum, with CG associated with the greatest degree of clinical impairment. Further supporting this notion, recent findings have provided evidence that the sense of a loss of control over eating is a transdiagnostic feature of several disordered eating behaviours (e.g., binge-eating, grazing, night eating, emotional eating) in both clinical and non-clinical populations [36]. Collectively, this highlights that the perceived loss of control over grazing is the key clinical feature associated with clinical impairment and should be assessed and prioritised in treatment. 

## 8. Limitations and Strengths

The findings of this study should be considered in the context of several limitations. First, psychological difficulties were assessed using self-report questionnaires rather than structured or semi-structured diagnostic assessments. Similarly, the use of self-report measures to ascertain grazing frequency may contribute to an overestimation in prevalence, as participants may overreport the frequency of their grazing. In addition, while useful in the screening of eating disorder psychopathology, the QEWP-5 has less utility in identifying BED and BN [17]. Consequently, this may have contributed to a misestimation in the number of participants presenting with eating disorders in this study. Second, this sample represented a more urbanised region of Brazil. As a result, findings may not equally generalise to less urbanised areas of Brazil. Importantly, previous research has found that Brazilian residents in rural areas have a lower prevalence of major depressive episodes than those in urban areas [37], which suggests potentially diverse psychological profiles across regions. Third, the cross-sectional design of this study limits our ability to determine causative mechanisms, and further research is required to extrapolate the causative pathways in grazing. Finally, although we had a relatively high participation rate, 23% of the eligible households declined to participate. Therefore, our results may have been influenced by non-response bias. 

In light of these limitations, there are several notable strengths. First, we utilised a large epidemiological sample that was representative of a major city in a middle-income country. Second, this was the first study to examine grazing and its clinical correlates in the general population of a middle-income country. Third, we utilised a robust and multi-stage epidemiological design, which attenuates the influence of non-response bias and strengthens the reliability of our findings. Fourth, we used a comprehensive and multi-method assessment procedure to identify BED and BN. In addition, we exclusively used validated psychometric measures to operationalise grazing (SIG), eating disorders and eating disorder symptomatology (QEWP-5), HRQoL (SF-12), depression (PHQ-9), and anxiety (GAD-7). Furthermore, anthropometric data was collected with standardised devices, which improves the accuracy of those measurements. Finally, we controlled for sociodemographic variances in our analyses, which minimised the impact of confounding variables on our outcomes [38].

## 9. Future Directions

While the findings of this study are noteworthy, there are several future directions for the field. First, future research should investigate the epidemiology of grazing in longitudinal studies. This may offer insight into the stability of grazing patterns across time, including whether certain factors contribute to shifting grazing from generic and innocuous to compulsive and disordered. Similarly, examining grazing patterns over time may provide an understanding of its contribution to increased body weight and eating disorder psychopathology. Second, future research should utilise ecological momentary assessment procedures to measure grazing in “real-time”. Collecting data in naturalistic environments may provide a greater understanding of the contexts (e.g., time, location, situation) in which grazing occurs as well as the individuals’ affective state (i.e., mood) before, during, and after grazing. This information is valuable considering that individuals may have difficulties accurately recalling their eating habits, potentially due to the automaticity of these behaviours [5,39,40]. Indeed, previous research has suggested that grazing may be conceptualised as an automatic and habitual behaviour [5,7,39,40], warranting further investigation into the role of habit and environmental triggers as the maintaining mechanisms of grazing. Relatedly, future studies should explore the role of executive function in grazing patterns, especially cognitive flexibility. Previous research has found that cognitive flexibility directly influences the strength of one’s eating habits [40], though this has not been explicitly investigated in grazing. Third, future studies should investigate grazing behaviours in low-income populations and different cultures as well as examining grazing patterns in children and adolescents. Finally, as proposed previously by Conceição et al. [6], a dimensional conceptualisation may be useful in providing greater nuance for these eating patterns, and to differentiate grazing from both OBE and SBE. 

## 10. Conclusions

This was the first, population-based, epidemiological study of grazing and its clinical correlates in a middle-income country. Overall, our findings indicated that the frequency of CG is consistent across middle- and high-income countries, while NCG appears to be less frequent in middle-income populations. In addition, similar to high-income populations, CG was associated with a higher BMI and higher odds of eating disorders, eating disorder symptomatology, depression, anxiety, and a lower physical and mental HRQoL than NG or NCG. Given the potential economic and health impacts of CG in middle-income countries, specialised public health services and training as well as flexible delivery of intervention (e.g., telemedicine) could promote equitable access to treatment. In addition, grazing patterns should be routinely assessed in clinical practice, especially for individuals presenting with an eating disorder and/or a high BMI.

## Figures and Tables

**Table 1:** Sociodemographic and weight characteristics according to grazing subtype.

Variable	NG (*n* = 1371)			NCG (*n* = 679)			CG (*n* = 239)			CG vs. NG	NCG vs. NG	CG vs. NCG
	*n*	%	95% CI	*n*	%	95% CI	*n*	%	95% CI	*p*	*p*	*p*
Gender										<0.001	0.269	<0.001
Women	803	48.3	[44.4, 52.3]	423	51.7	[47.4, 55.9]	176	71.9	[66.0, 77.0]			
Men	568	51.7	[47.7, 55.6]	256	48.3	[44.1, 52.6]	63	28.1	[23.0, 34.0]			
Age										0.007	0.009	0.711
18–30 years	294	28.4	[24.2, 33.0]	197	37.5	[31.7, 43.8]	76	40.6	[34.9, 46.6]			
31–45 years	480	38.1	[33.6, 42.8]	229	34.0	[29.2, 39.1]	87	33.4	[27.4, 40.1]			
46–60 years	597	33.5	[29.8, 37.5]	253	28.5	[24.7, 32.5]	76	25.9	[20.9, 31.7]			
Race/ethnicity										0.477	0.566	0.126
White	535	37.0	[33.1, 41.0]	288	39.2	[33.1, 45.6]	81	33.4	[26.6, 40.9]			
Black	240	19.8	[16.0, 24.1]	109	17.1	[13.6, 21.3]	52	24.6	[18.9, 31.2]			
Mixed	596	43.2	[39.9, 46.7]	282	43.7	[38.3, 49.2]	106	42.0	[32.7, 52.0]			
Marital status										0.071	0.355	0.333
Single	497	34.3	[30.3, 38.4]	263	37.1	[31.7, 43.0]	100	43.1	[33.6, 53.2]			
Married	660	54.9	[51.0, 58.7]	330	54.3	[48.8, 59.7]	110	49.9	[40.9, 59.1]			
Widow/divorced	214	10.9	[9.1, 12.9]	86	8.6	[6.5, 11.2]	29	6.9	[4.6, 10.3]			
Education										0.121	0.483	0.294
0–10 years	520	37.0	[31.4, 42.9]	246	34.8	[28.5, 41.8]	93	39.2	[32.0, 46.9]			
11–14 years	570	44.6	[39.9, 49.4]	303	48.9	[43.4, 54.5]	114	49.2	[41.7, 56.8]			
>15 years	281	18.5	[14.8, 22.8]	130	16.3	[11.9, 21.9]	32	11.6	[7.7, 17.1]			
Employment status										<0.001	0.008	0.004
Student	48	5.4	[3.5, 8.2]	42	8.4	[6.1, 11.6]	18	10.2	[6.1, 16.7]			
Paid employment	916	69.2	[65.8, 72.5]	413	59.2	[52.7, 65.4]	117	42.7	[35.9. 49.8]			
NIP employment	334	21.9	[18.8, 25.3]	198	30.0	[24.3, 36.5]	90	40.6	[34.4, 47.2]			
Retired	69	3.5	[2.5, 4.9]	26	2.3	[24.3, 36.5]	14	6.4	[3.6, 11.3]			
Income										0.098	<0.001	0.173
Up to R$1000	263	17.9	[14.6, 21.8]	186	34.5	[26.9, 43.1]	55	29.0	[19.5, 40.8]			
R$1001–3000	578	55.6	[50.3, 60.8]	228	39.8	[32.4, 47.8]	111	49.8	[41.2, 58.4]			
>R$3000	300	26.5	[21.1, 32.6]	135	25.6	[20.1, 32.1]	40	21.2	[13.6, 31.5]			
BMI class										<0.001	0.554	<0.001
Underweight	29	2.8	[1.5, 5.2]	25	4.0	[2.3, 6.8]	8	7.9	[3.3., 17.9]			
Normal weight	434	33.9	[28.9, 39.2]	204	31.4	[26.1, 37.2]	47	20.5	[15.1, 27.3]			
Overweight	503	36.6	[33.5, 39.9]	258	40.7	[32.8, 49.1]	60	24.0	[18.8, 30.1]			
Obesity	336	26.6	[23.2, 30.3]	158	24.0	[18.9, 30.0]	111	47.5	[41.3, 53.9]			
Obesity class										0.006	0.522	0.206
Class I	215	65.9	[57.0, 73.8]	98	65.3	[55.6, 73.8]	69	52.8	[36.8, 68.2]			
Class II	84	24.6	[18.1, 32.6]	37	21.5	[14.1, 31.4]	21	24.3	[14.4, 38.0]			
Class III	37	9.5	[6.6, 13.4]	23	13.2	[8.3, 20.3]	21	22.9	[15.2, 33.0]			
	**NG**			**NCG**			**CG**					
	***M* (*SE*)**	**95% CI**		***M* (*SE*)**	**95% CI**		***M* (*SE*)**	**95% CI**				
Age	39.32 (0.56)	[38.20, 40.43]		36.49 (0.70)	[35.10, 37.88]		36.29 (0.68)	[34.95, 37.62]		<0.001	<0.001	0.846
BMI	27.38 (0.26)	[26.87, 27.90]		27.24 (0.29)	[26.67, 27.80]		29.86 (0.58)	[28.71, 31.00]		<0.001	0.690	<0.001

Note. Missing values not included. All percentages (%) were calculated using the weighted frequencies. The number of participants in each group (*n*) were calculated using the unweighted count. 1000 BRL is approximately one minimum wage (monthly) in Brazil. Completing 0–10 years of education is equivalent to elementary school, 11–14 years of education is equivalent to high school, and >15 years of education is equivalent to college level or above. BMI = body mass index; CG = regular compulsive grazing; CI = confidence interval; NCG = regular non-compulsive grazing; NG = no grazing; NIP = not in paid.

**Table 2:** Prevalence and odds of eating disorders and eating disorder symptomatology according to grazing subtype.

Variable	NG (*n* = 1371)	NCG (*n* = 679)	CG (*n* = 239)	CG vs. NG			NCG vs. NG			CG vs. NCG		
	*n (*%*)*	*n* (%)	*n* (%)	OR	95% CI	*p*	OR	95% CI	*p*	OR	95% CI	*p*
Eating disorders												
BED	11 (1.0)	3 (0.3)	15 (6.8)	6.99	[2.04, 23.94]	<0.001	0.31	[0.07, 1.38]	0.104	22.36	[5.65, 88.55]	<0.001
BN	3 (0.1)	2 (0.4)	12 (5.6)	71.46	[14.72, 347.02]	<0.001	4.64	[0.71, 30.19]	0.077	15.40	[2.73, 86.95]	<0.001
Disordered eating behaviours												
OBE	55 (4.6)	43 (6.7)	72 (28.9)	8.47	[3.98, 18.03]	<0.001	1.50	[0.90, 2.50]	0.113	5.64	[2.89, 11.02]	<0.001
SBE	91 (5.9)	112 (19.6)	92 (42.1)	11.48	[7.04, 18.72]	<0.001	3.87	[2.62, 5.70]	<0.001	2.97	[1.88, 4.69]	<0.001
Compensatory behaviours												
OBE	53 (27.6)	9 (53.9)	5 (41.6)	1.87	[0.33, 10.57]	0.469	3.07	[0.44, 21.31]	0.240	0.61	[0.38, 0.98]	0.042 *
SBE	16 (14.5)	16 (13.7)	24 (33.9)	3.04	[1.27, 7.25]	0.011	0.94	[0.29, 3.07]	0.917	3.23	[1.45, 7.23]	0.004
Body evaluation												
Wg/Sh	431 (30.5)	296 (45.6)	127 (51.8)	2.45	[1.67, 3.60]	<0.001	1.91	[1.45, 2.51]	<0.001	1.28	[0.94, 1.75]	0.115
Dissat	376 (25.5)	243 (35.9)	148 (57.9)	4.03	[2.82, 5.76]	<0.001	1.64	[1.13, 2.38]	0.010	2.46	[1.76, 3.42]	<0.001

Note. All percentages (%) were calculated using the weighted frequencies. The number of participants in each group (*n*) were calculated using the unweighted count. Compensatory behaviours for objective binge-eating and subjective binge-eating included a combination of vomiting, diuretics misuse, laxatives misuse, diet pills misuse, fasting, and/or overexercising. BED = binge-eating disorder; BN = bulimia nervosa; CG = regular compulsive grazing; CI = confidence interval; Dissat = dissatisfaction; NCG = regular non-compulsive grazing; NG = no grazing; OBE = objective binge-eating; OR = odds ratio; SBE = subjective binge-eating; Wg/Sh = weight/shape. * This effect became non-significant after controlling for sociodemographic differences.

**Table 3:** Prevalence and odds of the psychological difficulties according to grazing subtype.

Variable	NG (*n* = 1371)	NCG (*n* = 679)	CG (*n* = 239)	CG vs. NG			NCG vs. NG			CG vs. NCG		
	*n (*%*)*	*n* (%)	*n* (%)	OR	95% CI	*p*	OR	95% CI	*p*	OR	95% CI	*p*
Depression	103 (6.5)	90 (11.1)	85 (37.8)	8.74	[5.83, 13.09	<0.001	1.80	[1.15, 2.80]	0.010 *	4.87	[3.00, 7.90]	<0.001
Anxiety	99 (6.7)	104 (13.1)	88 (37.1)	8.22	[4.60, 14.67]	<0.001	2.10	[1.32, 3.32]	0.002	3.92	[2.50, 6.16]	<0.001

Note. All percentages (%) were calculated using the weighted frequencies. The number of participants in each group (*n*) were calculated using the unweighted count. Depression was identified using a cut-off score of ≥10 on the PHQ-9. Anxiety was identified using a cut-off of ≥10 on the GAD-7. CG = regular compulsive grazing; CI = confidence interval; NCG = regular non-compulsive grazing; NG = no grazing; OR = odds ratio. * This effect became non-significant after controlling for sociodemographic differences.

**Table 4:** Means, standard error, and 95% confidence intervals for the health-related quality of life according to grazing subtype.

Variable	NG (*n* = 1371)		NCG (*n* = 679)		CG (*n* = 239)		CG vs. NG	NCG vs. NG	CG vs. NCG
	*M* (*SE*)	95% CI	*M* (*SE*)	95% CI	*M* (*SE*)	95% CI	*p*	*p*	*p*
PCS	54.20 (0.25)	[53.72, 54.69]	53.64 (0.41)	[52.82, 54.46]	50.75 (0.65)	[49.47, 52.03]	<0.001	0.178	<0.001
MCS	48.23 (0.36)	[47.52, 48.94]	44.85 (0.45)	[43.96, 45.76]	39.33 (0.97)	[37.41, 41.25]	<0.001	<0.001	<0.001
Overall	102.43 (0.49)	[101.46, 103.41]	98.50 (0.70)	[97.11, 99.88]	90.08 (0.92)	[88.26, 91.91]	<0.001	<0.001	<0.001

Note. Significance is based on results of individual t-tests. These effects remained significant after controlling for sociodemographic differences. Overall health-related quality of life is a composite of the physical component summary and mental component summary. CG = regular compulsive grazing; CI = confidence interval; MCS = mental component summary; NCG = regular non-compulsive grazing; NG = no grazing; PCS = physical component summary.

## Data Availability

The data presented in this study are available on request from the corresponding author. The data are not publicly available as this project is ongoing.

## Supplementary Materials

The following supporting information can be downloaded at: https://www.mdpi.com/article/10.3390/nu15030557/s1, Table S1: Additional test statistics.
