# Supplementary material for: Grazing Prevalence and Associations with Eating and General Psychopathology, Body Mass Index, and Quality of Life in a Middle-Income Country
Source: Nutrients. 2023 Jan 20;15(3):557. doi: 10.3390/nu15030557 (PMC9919254; doi:10.3390/nu15030557)
Supplement: Supplementary file 1 [file nutrients-15-00557-s001.zip › nutrients-2119487-supplementary.pdf]

Supplementary Table S1. Additional statistical tests.

Variable	CG vs. NG					NCG vs. NG					CG vs. NCG				
	Adj *F*	Df 1	Df 2	OR	*p*	Adj *F*	Df 1	Df 2	OR	*p*	Adj *F*	Df 1	Df 2	OR	*p*
Gender	46.71	1	120	2.73	< .001	1.23	1	120	1.14	.269	31.46	1	120	2.39	< .001
Age	5.42	1.7	208.5		.007	4.81	2.0	236.4		.009	0.31	1.8	214.9		.711
Race/ethnicity	0.72	1.8	217.4		.477	0.56	1.9	228.9		.556	2.11	1.9	228.6		.126
Marital status	2.88	1.6	188.2		.071	1.02	1.8	217.2		.355	1.08	1.7	207.3		.333
Education	2.14	2.0	234.3		.121	0.72	1.9	231.2		.483	1.23	2.0	237.8		.294
Employment status	15.14	2.4	285.8		< .001	4.23	2.6	316.4		.008	5.34	2.2	269.1		.004
Income	2.46	1.7	201.4		.098	10.60	1.9	230.2		< .001	1.77	1.9	232.5		.173
BMI class	8.67	2.0	236.3		< .001	0.64	2.4	286.5		.554	8.37	2.5	304.4		< .001
Obesity class	5.51	1.8	216.0		.006	0.64	1.9	225.3		.522	1.61	1.8	210.2		.206
Eating disorders															
BED	12.96	1	120	6.99	< .001	2.69	1	120	0.31	.104	40.48	1	120	22.36	< .001
BN	104.39	1	120	71.46	< .001	3.19	1	120	4.64	.077	17.07	1	120	15.40	< .001
Disordered eating behaviours															
OBE	40.87	1	120	8.47	< .001	2.55	1	120	1.50	.113	30.49	1	120	5.64	< .001
SBE	131.22	1	120	11.48	< .001	52.54	1	120	3.87	< .001	23.18	1	120	2.97	< .001
Compensatory behaviours															
OBE	0.53	1	120	1.87	.469	1.40	1	120	3.07	.240	4.24	1	120	0.61	.042 *

SBE	6.71	1	120	3.04	.011	0.01	1	120	0.94	.917	8.78	1	120	3.23	.004
Body evaluation															
Wg/Sh	22.07	1	120	2.45	< .001	22.07	1	120	1.91	< .001	2.52	1	120	1.28	.115
Dissat	65.40	1	120	4.03	< .001	6.91	1	120	1.64	.010	29.69	1	120	2.46	< .001
Depression	144.14	1	120	8.74	< .001	6.94	1	120	1.80	.010 *	46.70	1	120	4.87	< .001
Anxiety	65.42	1	120	8.22	< .001	10.51	1	120	2.10	.002	38.83	1	120	3.92	< .001

	**CG vs. NG**				**NCG vs. NG**				**CG vs. NCG**			
	**Wald *F***	**Df**	**Estimate**	***p***	**Wald *F***	**Df**	**Estimate**	***p***	**Wald *F***	**Df**	**Estimate**	***p***
BMI	14.24	120	2.473	< .001	0.160	120	−0.146	.690	18.70	120	2.619	< .001
Age	14.30	120	−3.033	< .001	12.94	120	−2.830	< .001	0.04	120	−0.203	.846
PCS	22.21	120	−3.453	< .001	1.83	120	−0.567	.178	13.07	120	−2.887	< .001
MCS	62.97	120	−8.897	< .001	31.72	120	−3.373	< .001	35.58	120	−5.524	< .001
Overall QoL	120.56	120	−12.350	< .001	22.92	120	−3.940	< .001	73.64	120	−8.411	< .001

Note. The adjusted *F* is a variant of the second-order Rao–Scott-adjusted chi-square statistical test. Significance is based on the adjusted *F* and its degrees of freedom. Depression was identified using a cut-off score of ≥10 on the PHQ−9. Anxiety was identified using a cut-off of ≥10 on the GAD−7. The overall health-related quality of life is a composite of the physical component summary and mental component summary. Adj = adjusted; BED = binge-eating disorder; BN = bulimia nervosa; BMI = body mass index; CG = regular compulsive grazing; Dissat = dissatisfaction; Df = degrees of freedom; MCS = mental component summary; NCG = regular non-compulsive grazing; NG = no grazing; OBE = objective binge-eating; OR = odds ratio; PCS = physical component summary; QoL = quality of life; SBE = subjective binge-eating; Wg/Sh = weight/shape. * This effect became non-significant after controlling for sociodemographic differences.
